# Lack of Bcr and Abr Promotes Hypoxia-Induced Pulmonary Hypertension in Mice

**DOI:** 10.1371/journal.pone.0049756

**Published:** 2012-11-12

**Authors:** Min Yu, Dapeng Gong, Min Lim, Anna Arutyunyan, John Groffen, Nora Heisterkamp

**Affiliations:** 1 Section of Molecular Carcinogenesis, Division of Hematology/Oncology, and The Saban Research Institute of Children’s Hospital, Los Angeles, California, United States of America; 2 Departments of Pediatrics and Pathology, Keck School of Medicine, University of Southern California, Los Angeles, California, United States of America; Vanderbilt University Medical Center, United States of America

## Abstract

**Background:**

Bcr and Abr are GTPase activating proteins that specifically downregulate activity of the small GTPase Rac in restricted cell types *in vivo*. Rac1 is expressed in smooth muscle cells, a critical cell type involved in the pathogenesis of pulmonary hypertension. The molecular mechanisms that underlie hypoxia-associated pulmonary hypertension are not well-defined.

**Methodology/Principal Findings:**

*Bcr* and *abr* null mutant mice were compared to wild type controls for the development of pulmonary hypertension after exposure to hypoxia. Also, pulmonary arterial smooth muscle cells from those mice were cultured in hypoxia and examined for proliferation, p38 activation and IL-6 production. Mice lacking Bcr or Abr exposed to hypoxia developed increased right ventricular pressure, hypertrophy and pulmonary vascular remodeling. Perivascular leukocyte infiltration in the lungs was increased, and under hypoxia *bcr−/−* and *abr−/−* macrophages generated more reactive oxygen species. Consistent with a contribution of inflammation and oxidative stress in pulmonary hypertension-associated vascular damage, Bcr and Abr-deficient animals showed elevated endothelial leakage after hypoxia exposure. Hypoxia-treated pulmonary arterial smooth muscle cells from Bcr- or Abr-deficient mice also proliferated faster than those of wild type mice. Moreover, activated Rac1, phosphorylated p38 and interleukin 6 were increased in these cells in the absence of Bcr or Abr. Inhibition of Rac1 activation with Z62954982, a novel Rac inhibitor, decreased proliferation, p38 phosphorylation and IL-6 levels in pulmonary arterial smooth muscle cells exposed to hypoxia.

**Conclusions:**

Bcr and Abr play a critical role in down-regulating hypoxia-induced pulmonary hypertension by deactivating Rac1 and, through this, reducing both oxidative stress generated by leukocytes as well as p38 phosphorylation, IL-6 production and proliferation of pulmonary arterial smooth muscle cells.

## Introduction

*BCR* (breakpoint cluster region) was originally identified as a gene involved in the development of Ph-chromosome-positive leukemias [1]. The normal Bcr protein of around 160-kDa contains multiple domains. One of these has an enzymatic function that is shared with Abr, a related protein encoded by a separate gene. The function of this domain has been relatively well characterized *in vivo*. It interacts with the small GTPase Rac when Rac is in its active GTP-bound conformation [2], [3]. The interaction of Bcr and Abr with RacGTP results in the conversion of bound GTP to GDP and the inactivation of Rac. Thus Bcr and Abr belong to the class of GTPase activating proteins (GAPs), of which around 70 have been identified for Rho family members [4]. Although many of these GAPs are expressed in a cell-specific or developmentally-restricted fashion, both Abr and Bcr are expressed in many cell types in an overlapping pattern.

There are only three Rac proteins, that regulate a diversity of biological functions. The expression of Rac2 and Rac3 is relatively restricted, whereas that of Rac1 is ubiquitous. Specificity of Rac function is regulated through a tightly controlled cycle of activation and deactivation that is mediated by upstream activators, the guanine nucleotide exchange factors (GEFs), and through deactivation by the GAPs [5], [6].

Mice lacking Bcr or Abr are phenotypically normal and fertile. However, when they are examined in more detail under conditions that generate pathology, significant differences with control wild type (WT) animals can be measured in specific functions. We found that ablation of Bcr and Abr results in abnormal reactivity of the innate immune system, and mice without both Abr and Bcr function also exhibit neuronal defects and inner ear developmental abnormalities [7]–[11].

Rac1 is also expressed in smooth muscle cells, which contribute to the pathology of pulmonary hypertension (PH), a life-threatening disorder [12]. Pulmonary vascular remodeling is a critical pathological feature of PH, and this is associated with the dysfunction and uncontrolled proliferation of the pulmonary arterial vascular smooth muscle cells (PASMCs) in the vascular wall. This in turn leads to increased thickening and muscularization of the small pulmonary arteries [13], [14]. We therefore considered the possibility that Abr and Bcr could regulate Rac activity in this cell type and through this mechanism affect the pathology of hypoxia-generated pulmonary hypertension. Using mice deficient in Abr or Bcr function, we here demonstrate for the first time that endogenous Rac1 activity is elevated by hypoxia-induced PH and that this is controlled by Bcr and Abr.

## Results

### Increased Hypoxia-induced Pulmonary Hypertension (PH) and Right Ventricle Hypertrophy in *bcr−/−* or *abr−/−* Mice

**Figure 1:**
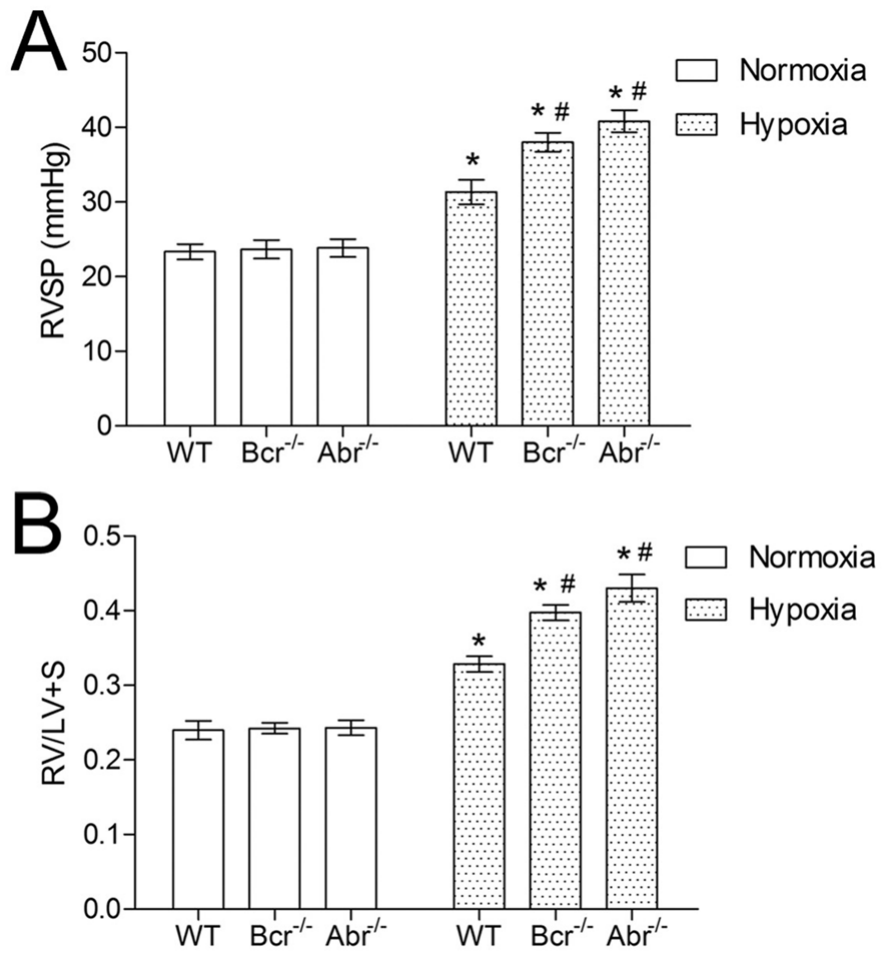
Hypoxia-treated *bcr−/−* and *abr−/−* mice have higher RVSP and more severe right ventricular hypertrophy. A, RVSP from *bcr−/−, abr−/−* and *wt* mice with exposure to normoxia or hypoxia. **B,** Ratio of RV/LV+S calculated using the weight of RV, LV+S from the hearts of normoxic and hypoxic *bcr−/−, abr−/−* and *wt* mice. *p<0.05 compared to the values of mice with the same genotype at normoxia. # p<0.05 when compared to *wt* mice in the same exposure condition. Bars represent mean ±SD; n  = 6 mice per group.

To examine the impact of chronic hypoxia on pulmonary hemodynamics in mice lacking Abr or Bcr, we measured right ventricular systolic pressure (RVSP). As illustrated in Figure 1A, the RVSP of *bcr−/−* or *abr−/−* mice was similar to that of *wt* mice in normoxia. After 3 weeks of exposure to hypoxia, all three genotypes developed pulmonary hypertension, with a significant increase in RVSP, compared with their littermates in normoxia. In the hypoxic *bcr−/−* or *abr−/−* mice, the RVSP was significantly higher than that in the hypoxic *wild type* (*wt)* mice (Figure 1A).

We next calculated the ratio of RV to LV+S weight, to assess the impact of changes of pulmonary pressure on cardiac mass (Figure 1B). There was no significant difference in (RV/LV+S) between normoxic *bcr−/−*, *abr−/−* and *wt* mice. Consistent with the RVSP, hypoxia increased the value of (RV/LV+S) in all mice, indicating the development of right ventricular hypertrophy. Compared with the hypoxic *wt* mice, the (RV/LV+S) value of hypoxic *bcr−/−* or *abr−/−* mice was higher, implying that the right ventricular hypertrophy was greater in these mice.

### Enhanced Hypoxia-induced Pulmonary Vascular Remodeling in *bcr−/−* or *abr−/−* Mice

**Figure 2:**
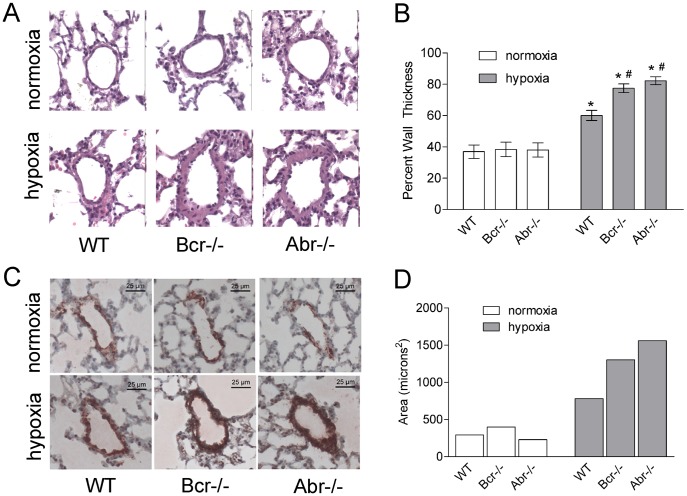
Hypoxia-induced pulmonary vascular remodeling in *bcr−/−* and *abr−/−* mice. **A,** Hematoxylin and eosin staining on representative lung specimens from *bcr−/−, abr−/−* and *wt* mice under normoxia or hypoxia. Note that the walls of the pulmonary arteries of the *bcr−/−* and *abr−/−* mice are remarkably thicker than those of the *wt* mice after hypoxia. Magnification, 200×. **B,** Quantification of changes in the pulmonary artery walls. Percent wall thickness was determined on H&E stained sections as described in Methods on nine vessels of comparable size per mouse, with 6 mice per genotype per condition. *p<0.05 compared with the same genotype at normoxia. # p<0.05 compared with WT mice in the same exposure condition. Bars, mean ± SD. **C,** Immunostaining with α-SMA antibodies on pulmonary vessels from representative normoxia or hypoxia-treated mice. **D,** Quantification of areas for α-SMA-positive cells. Areas of α-SMA-positive cells were calculated using ImageJ software as described in the Materials and Methods.

To investigate the pathologic changes in hypoxia-induced PH, we performed histological analysis on the lungs of these animals. In normoxia, the percent wall thickness was similar for the *bcr−/−*, *abr−/−* and *wt* mice. However, *bcr−/−* and *abr−/−* mice exposed to hypoxia showed significantly greater increase in the wall thickness and larger percent wall thickness, compared with the *wt* mice (Figure 2A and B). Immunostaining with α-SMA antibodies on lung specimens from the mice showed, as expected, that hypoxia-challenged mice showed pulmonary vascular remodeling, characterized by increased areas of α-SMA–immunoreactive PASMCs in the pulmonary arteries. After chronic hypoxia exposure, the *bcr−/−* and *abr−/−* mice developed visibly more severe vascular remodeling, with greater areas of α-SMA–positive cells in remodeled arteries than the *wt* mice (Figure 2C and D).

### Contribution of Inflammation to Hypoxia-generated Pulmonary Pathology in *bcr−/−* and *abr−/−* Mice

**Figure 3:**
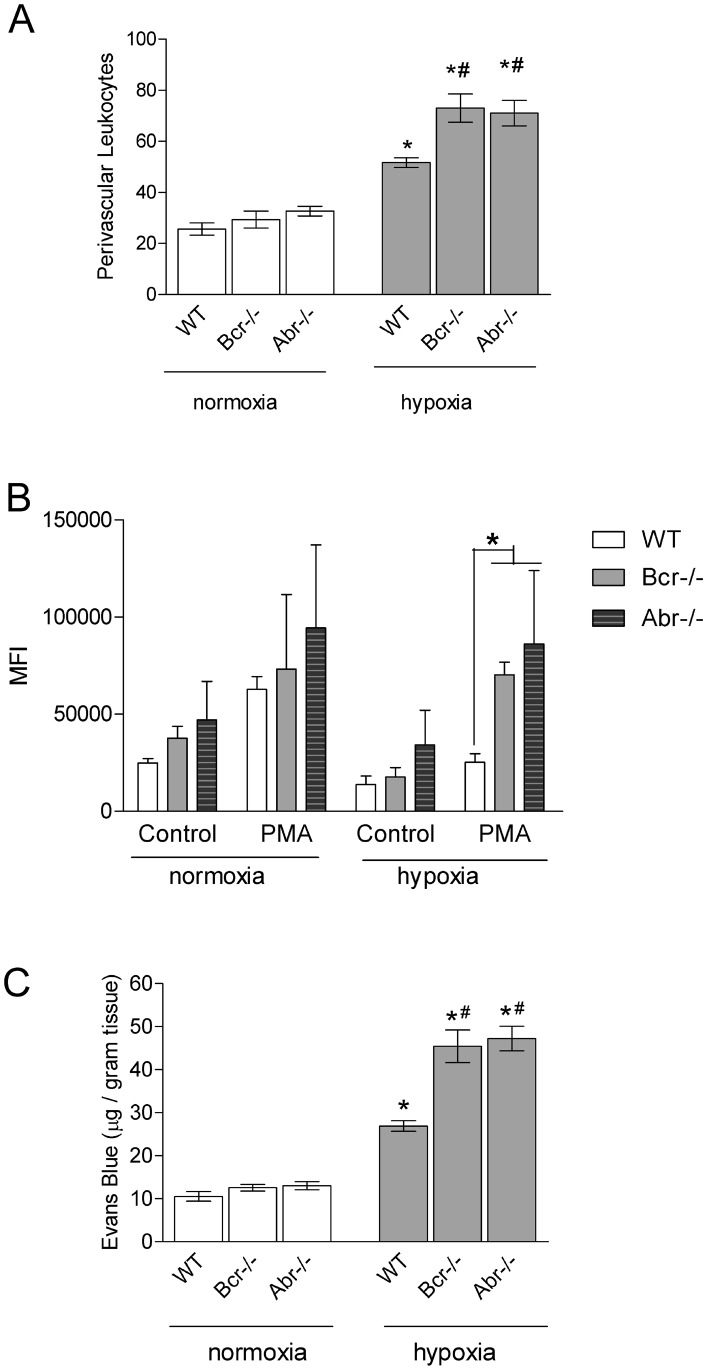
Lack of Abr and Bcr function exacerbates hypoxia-induced leukocyte involvement in PH. A, Numbers of perivascular leukocytes surrounding terminal arterioles. Data are expressed as total leukocyte counts per high-power field (200× amplification). Bars, mean+SEM. n = 3/group. **B,** Peritoneal-elicited macrophages exposed to normoxia or hypoxia for 24 hours activated with 1 µM PMA. ROS production was measured by FACS 1 hr later. Controls, cells without PMA activation. Bars, mean+SEM. n  = 5 mice/group. *, p<0.05. MFI, mean fluorescent intensity. **C,** Vascular leakage. Evans blue was injected when mice had been exposed to normoxia or hypoxia for 3 weeks. Bars, mean+SEM. n = 3–4 mice/group. A and C**,** *p<0.05 comparison between animals of the same genotype at normoxia. #p<0.05 compared to WT mice exposed to the same condition.

Inflammation and oxidative stress are thought to contribute to the pathology of PAH [15], [16]. Since Abr and Bcr negatively regulate Rac in innate immune cells [8], we quantitated the numbers of perivascular leukocytes in H&E stained lung sections. Interestingly, pulmonary extravasation of leukocytes under hypoxia was significantly increased in the mice lacking Abr or Bcr function as compared to *wt* mice (Figure 3A). To examine if reactive oxygen species (ROS) production could contribute to the exacerbated pathology in hypoxia-treated *bcr−/−* and *abr−/−* animals, we exposed macrophages from these mice to hypoxia and normoxia, and compared ROS production in their innate immune cells. We found that lack of these GAPs for Rac resulted in significantly increased ROS production under hypoxic conditions (Figure 3B). Excess ROS production by inflammatory cells can result in collateral damage to surrounding tissues including the endothelium. To address whether loss of Abr or Bcr function results in vascular injury, we exposed mice to hypoxia and then measured pulmonary endothelial dysfunction using Evans blue. Figure 3C shows that chronic hypoxia promoted loss of endothelial barrier function and, moreover, mice lacking Abr or Bcr were clearly more severely affected.

The VEGFR inhibitor SU5416 can cause emphysema in rodents and, when combined with chronic hypoxia, generates PAH [17], [18]. Pathological features of combined treatment with SU5416 and chronic hypoxia include increased endothelial cell proliferation and moderately increased emphysema compared to mice exposed to hypoxia alone [19]. However, mice and rats with PAH induced by hypoxia alone did not show increased proliferation of pulmonary artery endothelial cells [20]. We therefore examined lungs of mice lacking Abr or Bcr for signs of emphysema. No obvious differences in airway hypertrophy between genotypes exposed to hypoxia were noted (Figure S1A). The mean linear intercept and average alveolar area in *bcr−/−* and *abr−/−* mice was increased upon chronic hypoxia exposure, but this was not significantly different from that of mice at normoxia, or of *wt* mice at hypoxia (Figure S1B, C).

### Proliferation of *bcr−/−* and *abr−/−* PASMCs is Stimulated by Hypoxia

**Figure 4:**
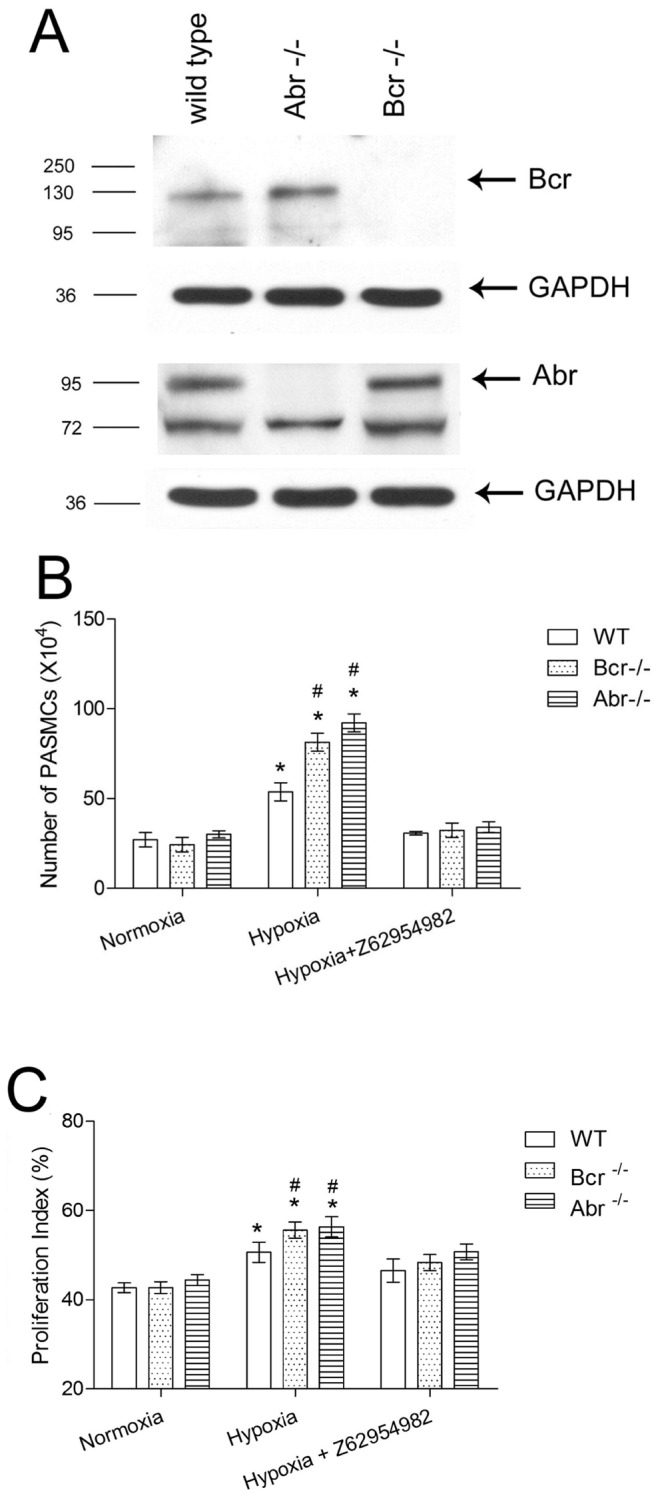
*Bcr−/−* and *abr−/−* PASMC show increased proliferation when exposed to hypoxia *in vitro*. A, Western blot analysis on PASMC lysates from *wt*, *bcr−/−* or *abr−/−* mice with anti-Bcr N20 antibodies or Abr antiserum. GAPDH, loading control. **B,** Third passage primary PASMC isolated from the intrapulmonary arteries of 5 different mice per genotype (1×10^4^ cells/well) were synchronized by serum free medium for 24 hrs, then cultured in medium with 10% FBS for 5 days, after which cells were counted. The Rac inhibitor Z62954982 was added to the indicated samples. * p<0.05 compared with the outcomes from the same genotype PASMCs in normoxia. # p<0.05 compared with *wt* PASMCs exposed to the same condition. Bars, mean ±SD of triplicate wells. **C,** Proliferation index of *wt*, *bcr−/−* and *abr−/−* PASMCs was calculated as described in Methods. Flow cytometry analysis was done based on 10,000 PASMCs per sample, 3 samples per genotype per condition. * p<0.05 compared with the outcomes from the same genotype PASMCs in normoxia. # p<0.05 compared with *wt* PASMCs exposed to the same condition. Bars are shown as mean ±SD of triplicate wells.

The progress of pulmonary vascular remodeling not only involves endothelial dysfunction, but also adventitial fibroblast growth, matrix deposition, acquisition of new mural cells and PASMCs proliferation [21]. These result in the narrowing of the lumen and decreased compliance of the small pulmonary arteries, constituted mainly by PASMCs [22], [23]. To examine if Bcr and Abr are expressed in PASMCs, we performed Western Blot analysis. As shown in Figure 4A, both are expressed in this cell type. To explore the underlying mechanism of the enhanced pulmonary vascular remodeling in *bcr−/−* and *abr−/−* mice, we investigated the *in vitro* proliferation of PASMCs from these animals. As shown in Figure 4B, PASMCs from the different genotypes had similar proliferation in normoxia. The proliferation of all PASMCs increased under hypoxic conditions; furthermore, the proliferation of *bcr−/−* or *abr−/−* PASMCs was significantly higher than that of the *wt* cells.

We also investigated the effect of a novel Rac inhibitor, Z62954982, on the proliferation of the PASMCs. This compound has been shown to inhibit Rac but not RhoA or CDC42 activation in SMC [24]. Interestingly, Z62954982 clearly suppressed the proliferation of hypoxia-treated PASMCs, reducing it to that measured for normoxia. Flow cytometry cell cycle analysis confirmed that the hypoxic condition induced an increase in the PASMC proliferation as quantified by the proliferation index, which was more significantly elevated in PASMCs that lacked Bcr or Abr, and could be decreased by Z62954982 (Figure 4C).

### Deficiency of Bcr or Abr Enhances Rac Activity during Hypoxia

**Figure 5:**
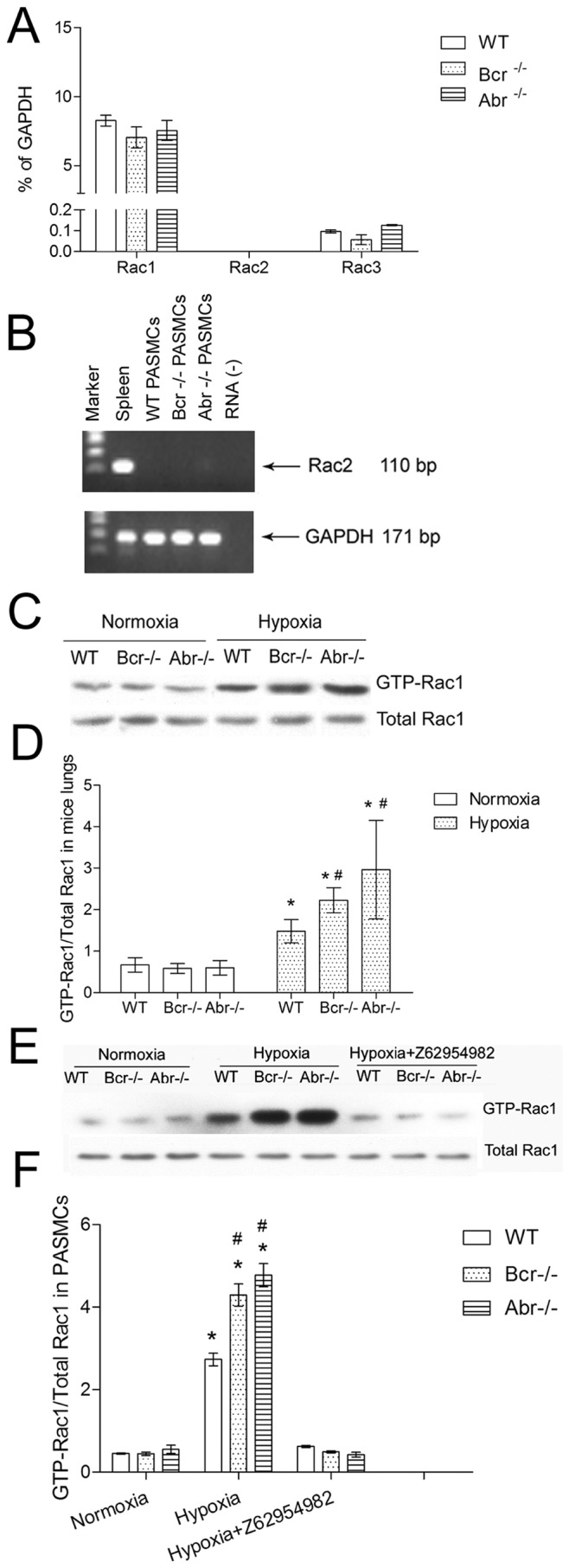
Loss of Bcr or Abr promotes hypoxia-induced Rac1 activation *in vitro* and *in vivo*. A, Real-time RT-PCR analysis for quantification of *rac1*, *rac2* and *rac3* mRNA in PASMCs. **B,** Representative gel electrophoresis of RT-PCR products showing absence of *rac2* in PASMCs. Samples loaded are indicated above the lanes; spleen, positive control; RNA (−), no RNA, negative control. **C–F,** Assay for Rac1 activation. **C–D,** Analysis of activation of Rac1 *in vivo* in the lungs of *bcr−/−*, *abr−/−* and *wt* mice after normoxia or hypoxia exposure. **E–F,** Analysis of Rac1 activation in PASMC under normoxia or hypoxia. **C** and **E,** representative Western blots; **D** and **F,** quantification. Three independent samples of each genotype were tested and the entire experiment was repeated independently. To quantitate results, Western blots were scanned and the ratio of GTP-Rac/total Rac was determined (panels **D, F**). * p<0.05 when compared with the results of the same genotype under normoxia. # p<0.05 when compared with WT exposed to the same condition.

Because Abr and Bcr can regulate all three Racs we used quantitative real-time PCR to investigate which Racs are expressed in PASMCs. As shown in Figure 5A and 5B, *rac1* is highly expressed, *rac2* mRNA can not be detected, and *rac3* expression is very low in this cell type.

Regulation of Rac expression levels can be one mechanism to control the effect of Rac, but it is generally accepted that the most important mode of regulation is on its GTP- or GDP-bound state. In macrophages, astroglia and neutrophils, loss of Abr and Bcr function leads to increased levels of activated Rac [7]–[9], [11]. Therefore, we examined the activation state of Rac1 protein in lungs from normoxic and hypoxic mice and in PASMCs. As shown in Figure 5C, the baseline levels of GTP-Rac1 (active Rac1) were similar in the lungs of normoxic *bcr−/−*, *abr−/−* and *wt* mice. Hypoxia increased the level of GTP-Rac1 in all lung samples. Interestingly, the level of GTP-Rac1 was higher in the lungs from hypoxic Bcr−/− or Abr−/− mice than in those of the WT mice (Figure 5C and D). In PASMCs, a similar basal level of GTP-Rac1 in different genotypes in normoxia was measured (Figure 5E, left 3 lanes). Hypoxia increased the levels GTP-Rac1 in PASMCs and this was higher in *bcr−/−* or *abr−/−* PASMCs than *wt* controls (Figure 5E, 5F). We also measured active Rac in hypoxia-treated PASMCs exposed to the Rac inhibitor Z62954982, and found that it significantly inhibited activation of Rac1.

To investigate the possible application of this compound *in vivo,* we treated *abr−/−* mice with intraperitoneal injections of 10 mg/kg, every second day for the duration of the hypoxia exposure. Analysis of RV/LV+S revealed that the right ventricle hypertrophy index was decreased in Z62954982-treated *abr−/−* mice under hypoxia but this was not statistically significant. The trend was more obvious in an experiment in which 20 mg/kg/d was used (p = 0.096) (Figure S2A). There was no evidence for hepatic toxicity (10 mg/kg, Figure S3A) or myelosuppression at 20 mg/kg (Figure S3B). Lung lysates isolated 60 minutes after application of the final lower dose of Z62954982 showed that Rac activation was inhibited at least temporarily in this target tissue (Figure S2B, C).

### Lack of Bcr or Abr Promotes Phosphorylation of p38 MAPK and Increased IL-6 in Hypoxia

**Figure 6:**
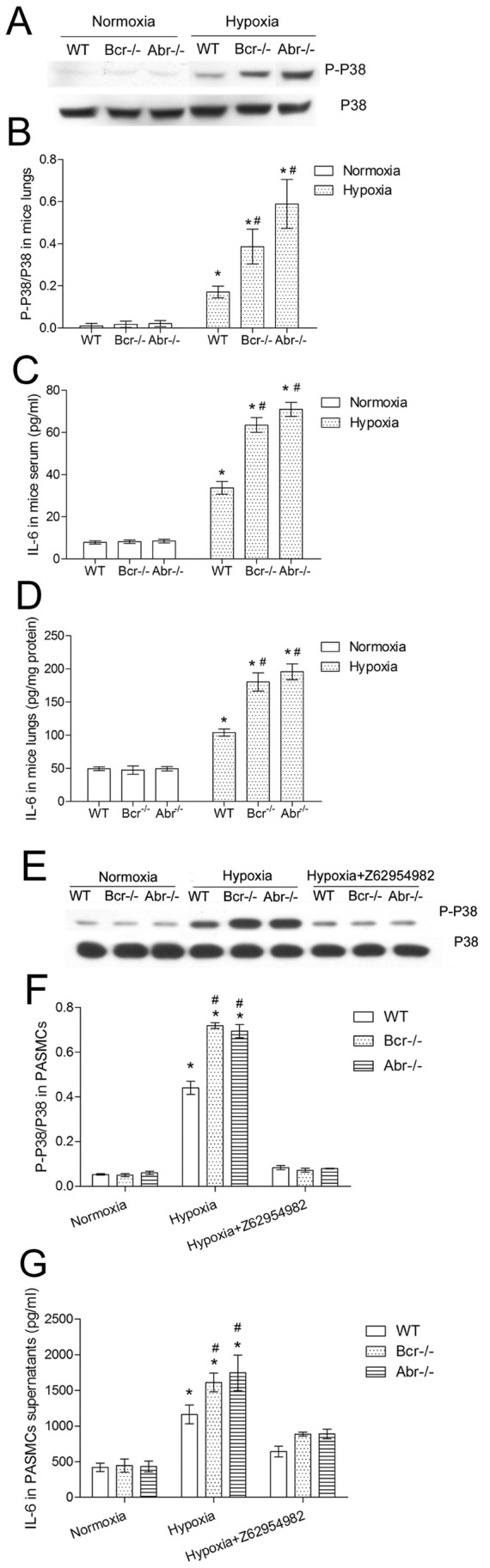
Enhanced phosphorylation of p38 and IL-6 production *in vivo* and in null mutant PASMC. A, Representative image of Western blots analyzing the phosphorylation of p38 in the lungs from *bcr−/−*, *abr−/−* and *wt* mice. **B,** Quantification of results for lungs from 6 mice/group. **C–D,** IL-6 was measured using an ELISA in serum (**C**) and lungs (**D**) of *bcr−/−*, *abr−/−* and *wt* mice using 6 independent samples per genotype per condition. **E–F,** Western blot analysis of PASMC for p-p38 (**E**) and quantification (**F**) of results of 3 independent samples of PASMCs/group. **G,** IL-6 in PASMC supernatants after normoxia or hypoxia exposure. *p<0.05 compared with the values from the same genotype, or PASMCs in normoxia. #p<0.05 when compared with WT exposed to the same condition.

Among the various intracellular pathways that can be activated by Rac, the p38 MAPK pathway was reported to be specially relevant to the pathological changes that occur in pulmonary hypertension [25]–[28]. Also, p38 activation was found to be associated with increased IL-6 production [29]–[37]. To investigate whether the p38 kinase pathway and concomitant IL-6 production are involved in the Bcr or Abr deficiency-promoted pulmonary hypertension, activation of the p38 pathway in the lungs and IL-6 levels in the serum and lungs of these mice were assessed. Under conditions of normoxia, the phosphorylation of p38 was minimal (Figure 6A, B) and IL-6 levels were low in serum and in lungs (Figure 6C, D). Exposure to hypoxia remarkably elevated the levels of p-p38 in lungs and this was increased significantly more in *bcr−/−* or *abr−/−* mice than in the *wt* mice (Figure 6A, B). This was accompanied by a significantly larger increase in IL-6 levels in lung and serum in the null mutant mice (Figure 6C, D).

We also evaluated p38 activation and IL-6 production in the PASMCs. When present at normoxia, PASMC had very low levels of p-p38 and produced little IL-6, but after hypoxia exposure this was significantly increased (Figure 6E–G), especially in *bcr−/−* or *abr−/−* PASMCs. To explore whether the Rac activation was correlated with the p38 MAPK phosphorylation and IL-6 production, we assessed p-p38 and IL-6 production in PASMCs treated with the Rac inhibitor Z62954982. Our results show that Z62954982 decreased both phosphorylation of p38 as well as secreted IL-6 in PASMCs in response to hypoxia (Figure 6E–G).

## Discussion

Pulmonary hypertension is a devastating disease with poor prognosis. Because no validated therapy has been reported that can prevent it or affect its development [12], [38], the identification of potential targets for treatment remains a crucial first step.

A number of studies indirectly suggest that Rac may play a role in PH and form such a potential target [39]–[42], but the possible contribution of endogenous Rac protein to PH has not been adequately examined. Firstly, since there are three distinct Rac proteins, Rac1, Rac2 and Rac3, there is a controversy regarding which of these are expressed in PASMCs, a critical cell type involved in the pathogenesis of PH. Although Rac2 is regarded as an isoform that is only expressed in hematopoietic cells, Rac2 was reported to also be present in aortic smooth muscle cells [43], [44]. However Rac2 expression in PASMCs had not been reported. In the current study, using quantitative real-time RT/PCR, PASMCs cultured in standard culture medium [45] were found to contain virtually no *rac2* mRNA. Our results additionally showed low *rac3* and high *rac1* mRNA levels in PASMC, indicating that Rac1 is likely to be the most important Rac for this cell type.

Secondly, previous reports that suggest Rac1 regulates processes relevant to pulmonary hypertension based their conclusions on the detection of increased levels of total Rac1 protein using Western blotting, or on the overexpression of constitutively active V12Rac or dominant negative N17Rac in PASMCs [40], [46]. Although the latter studies obviously are important, under physiological conditions Rac proteins are never permanently frozen into a GTP- or GDP-bound state, but instead participate in a cycle of activation and deactivation. Also, in our study, no evidence was found for increased total Rac1 protein levels upon exposure of mouse lungs or PASMC to hypoxia (Figure 5E). Therefore, we here analyzed the levels of endogenously activated, GTP-bound Rac1 as a physiologically relevant readout for the putative involvement of Rac in PH.

We were able to detect not only the activation of endogenous Rac1 in the lungs in a mouse model of PH, but we also found enhanced hypoxia-induced activation of pulmonary Rac1 in mice deficient of Bcr or Abr. Consistent with the *in vivo* results, we confirmed that Rac1 becomes activated in PASMCs that are subjected to hypoxia. Moreover, the hypoxia-induced activation of Rac1 was significantly more notable in PASMCs lacking Bcr or Abr. We conclude that Bcr and Abr are very important components of a regulatory pathway that connects PH caused by hypoxia to Rac activation and the proliferation of PASMC. Our data also connect Bcr and Abr to the pathology of PH in a different cell type, namely by suppression of excessive ROS production in macrophages exposed to hypoxia.

The activation of Rac1 in PASMC can lead to a number of essential signaling events. The phosphorylation of p38 has been reported as a critical factor in the development of pulmonary hypertension [47], [48]. Our results also showed for the first time that the suppression of endogenous Rac1 activation with the Rac inhibitor Z62954982 led to inhibition of phosphorylation of p38 in hypoxic PASMCs. This result is consistent with a previous study which reported that knock-down of Rac1 with siRNA suppresses the phosphorylation of p38 in aortic smooth muscle cells exposed to cyclic strain [49].

Of the various cytokines that are generated downstream of the phosphorylation and activation of the p38 pathway, IL-6 was recently highlighted because of its important role in pulmonary vascular remodeling and pulmonary hypertension [29], [30], [32]. In agreement with previous reports, our study showed increased IL-6 in mouse serum and lung homogenates in hypoxia-induced PH. We also observed further elevated IL-6 in hypoxic Bcr- or Abr-deficient mice. IL-6 can be secreted by vascular smooth muscle cells, and can also promote their growth [36], [50]. Moreover, hypoxia is a factor that induces transcription and translation of IL-6 in human PASMCs [51]. Our results are consistent with these reports. We confirmed that hypoxia can increase IL-6 levels in the culture supernatant of PASMCs. Interestingly, we showed that decreasing activated Rac1 levels with Z62954982 can decrease IL-6 produced by hypoxia-exposed PASMC, suggesting that IL-6 is regulated by Rac1 activation, and is possibly related with phosphorylation of p38.

**Figure 7:**
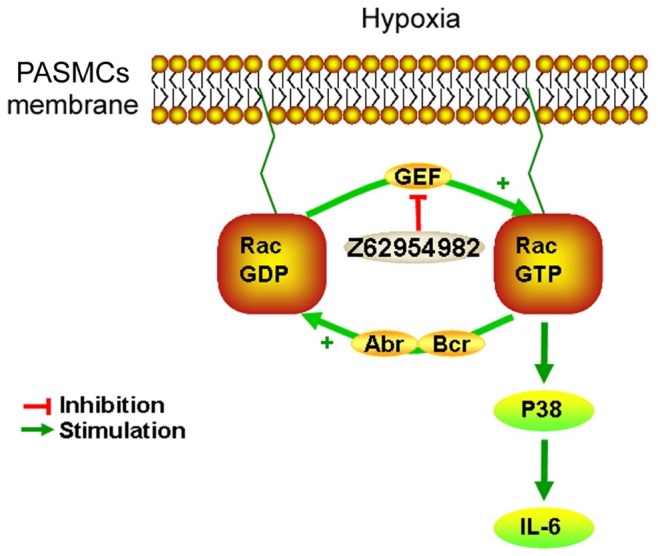
Schematic representation of the signal transduction pathways affected in PASMC lacking Abr and Bcr. Hypoxia directly or indirectly activates a G-nucleotide exchange factor (GEF) for Rac, resulting in elevated levels of GTP-bound Rac. In the absence of Bcr or Abr, Rac is not downregulated to its inactive GDP-bound form and this causes prolonged activation of p38 MAPK and production of IL-6. The inhibitor Z62954982 prevents the generation of the GTP-bound Rac that switches these pathways on, by interfering with the binding of Rac to the GEF that is chronically switched on in hypoxia.

Figure 7 summarizes our data that link Abr and Bcr to the Rac cycle and downstream events in hypoxia-treated PASMCs. Which GEF is upstream of Rac in promoting PASMC proliferation, and how that GEF is activated in PH, is currently unknown. We noted that different GEFs for Rac including *DOCK1*, *TIAM1, TRIO, VAV1*, *VAV2* are highly expressed both in human control lung as well as PH RNA samples (GSE15197) This does not support the possibility that the GEFs are regulated at the level of gene transcription. Instead, we suggest that the GEF may be activated through tyrosine phosphorylation. In particular, PDGF-R signaling is known to play a role in pulmonary hypertension [52]. Since hypoxia induces increased *PDGF-BB* mRNA in human primary VSMCs, which also express the PDGF-R, paracrine signaling may contribute to VSMC cell proliferation under hypoxic conditions [53]. PDGF signaling in VSMC also generates reactive oxygen species, leading to the activation of the Src and Abl tyrosine kinases and promoting growth of these cells [54]. In fibroblasts, Abl is needed for the PDGF-R signaling that leads to Rac activation and mitogenesis [55]. Dock1 (DOCK180), a GEF for Rac, becomes tyrosine phosphorylated by Src downstream of PDGFRα activation in glioblastoma cells [56]. Thus, we speculate that one mechanism through which hypoxia could result in Rac activation is by paracrine PDGF-R signaling, that in turn leads to ROS production, Src/Abl activation and tyrosine phosphorylation/activation of a GEF for Rac. This mechanism would be consistent with the activity of the Abl and PDGF-R inhibitor Imatinib currently in clinical trials for PAH.

There are very few small molecule inhibitors available that decrease levels of activated Rac [24]. Z62954982 is a new compound that was identified through pharmacophore virtual screening, with the commonly used Rac inhibitor NSC23766 as reference compound [24]. Z62954982 does not inhibit Rac directly, but interferes with the interaction of Rac with a GEF, which activates Rac by catalyzing the exchange of the GDP bound to it for GTP. Considering the fact that there are 69 GEFs in mammals that act on various GTPases with a probable tissue specific pattern of expression [57], the targets of such inhibitors of the GEF-Rac interaction are not well-defined and need to be empirically discovered.

Z62954982 was tested in human vascular smooth muscle cells, in which it interfered with the binding of Rac1 to Tiam1 and was more efficient in reducing Rac1-GTP levels than the chemically unrelated Rac inhibitor NSC23766 [24]. However, Z62954982 has not been used in animals. In our experiments, we measured reduction in levels of activated Rac and found evidence for a beneficial effect on the pathology of hypoxia-induced PH in mice. The relatively moderate extent of this effect could be caused by, for example, a rapid clearance of the drug, and further pharmacokinetic and pharmacodynamic studies will be needed to determine the utility of this particular chemical compound for treatment of PAH.

Our results do establish that lack of Abr and Bcr function, resulting in elevated levels of activated Rac, affects macrophages and PASMC, two distinct cell types that are involved in the pathology of PH. Thus treatment with Rac inhibitors remains a viable approach to suppress hypoxia-induced pulmonary hypertension, in particular if a combination of GEF inhibitors, or a single Rac inhibitor can be identified that would target Rac activation in PASMC as well as in innate immune cells.

## Materials and Methods

### Ethics Statement

Animal experiments were approved by the Children’s Hospital Los Angeles Institutional Animal Care and Use Committee, and were conducted in compliance with the NIH guide for the Care and Use of Laboratory Animals.

### Animals and Chronic Hypoxia Model

*Bcr−/−* and *abr−/−* mice were generated as previously reported [9], [11] and were on an f6 FVB/J inbred genetic background.

Male *bcr−/−*, *abr−/−* and *wt* mice (8 to 10-week-old littermates) were exposed to hypoxia (10% O_2_) or normoxia (21% O_2_) for 3 weeks. For *in vivo* Rac1 inhibitor treatment experiments, Z62954982 was administered intraperitoneally (i.p.) at 10 mg/kg every other day or 20 mg/kg daily. Control mice were injected with the same volume of vehicle (DMSO : corn oil  = 1∶ 9). During and at the end of the treatment, we monitored normoxia-exposed vehicle and Z62954982-treated wild type mice for possible obvious signs of toxicity of the drug. However there was no evidence for toxicity. Numbers of myeloid cells in the bone marrows were comparable and kidney (not shown) and liver appeared normal in both groups (Figure S3).

Mice were anesthetized with intraperitoneal ketamine hydrochloride (60 mg/kg) and xylazine (8 mg/kg). A catheter connected to a pressure transducer was inserted into the right ventricle (RV) through the right jugular vein. Right ventricular systolic pressure (RVSP) was measured by BP-1 (World Precision Instruments, FL, USA). The mice were then sacrificed with CO_2_. The serum, hearts and lungs were immediately collected.

### Pathology and Image Analysis

Percent wall thickness was calculated based on the analysis of the arterial area of pulmonary terminal arterioles, according to the following formula: wall thickness (%)  =  (area_ext_–area_int_)/area_ext_x100 (area_ext_ is the area bounded by external wall of the vessel, and area_int_ is the area bounded by internal wall of the vessel). Nine vessels of comparable size (25–100 µm) per mouse were measured (6 mice per group). In total, 324 vessels were included. PASMCs were detected by immunohistochemistry with a mouse monoclonal antibody to α-smooth muscle actin (α-SMA; 1∶1000, Sigma).

For assessment of right ventricle hypertrophy, right ventricles (RV), left ventricles and septae (LV+S) were isolated and weighed. The ratio of the weight of RV to LV+S (RV/LV+S) was analyzed as an index of right ventricle hypertrophy (RVH).

For examination of the lungs, the right primary bronchi of the lungs were ligated. The left lobes were inflated through the trachea with 10% formalin at a perfusion pressure of 20 cm H_2_O. Excised inflated lung tissue was fixed in 10% formalin, processed and embedded with paraffin. Hematoxylin and eosin staining was performed on 5 µm sections. The images of the pulmonary terminal arterioles were captured with a Zeiss microscope digital camera. The arterial areas were analyzed with ImageJ 1.41 software (NIH, Bethesda, MD) for percentage wall thickness.

For immunohistochemistry, lung sections (5 µm) were deparaffinized. Vascular smooth muscle cells were detected by immunohistochemistry with a mouse monoclonal antibody to SMA, using a LAB-SA detection system (Invitrogen). Areas of cells positive for anti-SMA staining were quantified using ImageJ software (NIH). Briefly, contour plots were drawn around the α-SMA-stained cells. Images were then converted to grayscale format. A threshold was set for α-SMA-positive staining. Within the contour plots, pixels with intensities above the threshold were quantified and converted to areas using the scale bar on the image.

### Measurement of the Mean Linear Intercept

For each image, 4 areas of interest, free of airways and blood vessels, were randomly picked for measuring the Mean Linear Intercept (MLI). A grid (5×5 with 20 µm between lines) was superimposed over the area of interest. The number of times (intercepts) that the alveoli intercepted the grid lines was counted. The MLI was calculated by multiplying the length of the lines and the number of lines per area, then dividing by the total number of intercepts [58].

The average alveolar area was quantified with Fiji ImageJ software [59]. The images were first processed with a median filter (radius 2.0 pixels), color deconvolution with an H&E 2 matrix, and an unsharp mask (radius 20 pixels, weight 0.9) on the H&E image. Default auto-thresholding and a binary fill-holes function were next applied. The area of all objects was measured with the Analyze Particles function, excluding objects with areas <60 µm^2^. Objects representing trachea and blood vessels were manually excluded from the analysis. Data represent >100 measurements per genotype per condition.

### Measurement of ROS Production in Macrophages

Mice were injected intraperitoneally with 4 ml of 4% thioglycollate medium. 4 days after the injection, macrophages were harvested from peritoneal cavities. Macrophages in complete RPMI-1640 medium (10% FBS) were exposed to normoxia or hypoxia for 24 hours. Cells were then treated with PBS (control cells for basal ROS production) or activated with PMA (1 µM) for 1 hr. Measurement of ROS production was performed using a CellROX Deep Red Reagent kit (Life Technologies, CA) according to the manufacturer’s protocol. Data were collected using an Accuri flow cytometer (BD Biosciences).

### Evans Blue Vascular Leakage Assay

Mice subjected to hypoxia for 3 weeks were injected with Evans blue (30 mg/kg body weight) via the tail vein. 20 min after the injection, animals were sacrificed and perfused with PBS through the right ventricle. The vena cava was cut to drain blood and PBS, thus allowing PBS to pass through the pulmonary and systemic circulation to flush out blood. After perfusion, lungs were harvested, blotted on gauze and weighted. Lungs were then placed in 1 ml of formamide and incubated at 56°C for 24 h for the extraction of Evans blue. The concentration of Evans blue was measured at OD620 and calculated against a standard curve.

### Pulmonary Arterial Smooth Muscle Cells Isolation and Culture

PASMCs were isolated from the intrapulmonary arteries of male *bcr−/−, abr−/−* and *wt* FVB/J mice as previously described [60]. In brief, mice were euthanized by CO_2_. The thorax and abdomen were rinsed with 70% ethanol. The intrapulmonary arteries were dissected with sterile scissors and forceps under microscope. The adventitia and intima were removed from the arteries. The arteries was cut into 1–2 mm long pieces and incubated with 1.5 mg/ml type II collagenase (Sigma) for 4–6 hours at 37°C with gentle shaking. After two washes with culture medium (DMEM with 10% fetal bovine serum, 1% penicillin/streptomycin, 1% glutamine; Invitrogen), PASMCs were collected and cultured at 37°C in 95% air/5% CO_2_. The PASMC phenotype was confirmed by morphological features and immunostaining for SMA.

### *In vitro* Cell Proliferation Assay and Flow Cytometric Cell Cycle Analysis

PASMCs (3^rd^ passage) from male *bcr−/−, abr−/−* and *wt* FVB/J mice were seeded in 6-well plates (1×10^4^ cells/well) and cultured in serum-free medium for 24 hrs. Effective cell synchronization was confirmed by flow cytometry analysis. Triplicate wells of cells were then exposed to normoxia or hypoxia (5% O_2_), with or without 25 µmol/L of the Rac inhibitor Z62954982 (ZINC08010136; Enamine Ltd, Cincinnati, USA). Z62954982 was made as a concentrated stock solution in DMSO (dimethyl sulfoxide). The final concentration of DMSO in the culture medium was 0.1%; control wells were treated with 0.1% DMSO only. Z62954982 did not have cytotoxic effects, since viability of the drug-treated PASMCs was around 95%, comparable with that of PASMCs treated with 0.1% DMSO. Medium was refreshed every 2 days. After 5 days, the supernatants were collected for measurement of IL-6. Cells were trypsinized and counted. PASMCs were then fixed with 70% ethanol and incubated with staining solution (20 µg/ml propidium iodide, 0.2 mg/ml DNase-free RNase, 0.01% Triton-100 in PBS). Cell cycle was analyzed using a flow cytometer (Accuri Cytometers). The proliferation index of the PASMCs was calculated as: (S+G_2_/M)/(G_0_/G_1_+ S + G_2_/M) ×100%. G_0_/G_1,_ S, G_2_/M represents the percentage of the cells in G_0_/G_1,_ S, G_2_/M phase, respectively.

### Quantitative Real-time PCR

For the isolation of RNA and protein, 3^rd^ passage PASMCs from Bcr−/−, Abr−/− and WT mice were plated in 10 cm culture dishes (3×10^4^/dish). Cells were treated either with DMSO or with Z62954982 (25 µmol/L, dissolved in DMSO, Enamine Ltd) in an incubator with normoxia or hypoxia (5% O_2_) for 5 days. Medium was refreshed every other day. Fresh Z62954982 was added along with the change of medium for the Z62954982 treatment cells. On day 5, cells were harvested, washed 3 times with PBS, harvested by scraping and processed for RNA (RNA mini kit, Qiagen) or for protein isolation in MLB (see below). 2 µg of RNA was converted to cDNA. The primer pairs used for amplification were:

*rac1*, 5'- TGGCGAAAGAGATCGGTGCTGT-3' (sense),

5'-TTCTTGACAGGAGGGGGACAGAGA-3' (antisense);

*rac2*, 5'-ACCTCCTAGCCACTCCATACCACT-3' (sense),

5'-CACCACACACTTGATGGCCTGCAT-3' (antisense);

*rac3*, 5'-TGAGAATGTCCGTGCCAAGTGGT-3' (sense),

5'-CCGCAGCCGTTCAATCGTATCCTT-3' (antisense);

Glyceraldehyde 3-phosphate dehydrogenase (*gapdh*),

5'-ACCCAGAAGACTGTGGATGG-3' (sense),

5'-CACCACACACTTGATGGCCTGCAT-3' (antisense).

Amplification reactions were performed with SYBR Green (Invitrogen) in an ABI Prism 7700 thermal cycler sequence detection system (Perkin-Elmer, CA). To confirm specificity of amplification, the PCR products from each primer pair were subjected to a melting curve analysis and electrophoresis on 2% agarose gels. The relative mRNA levels of target genes to that of *gapdh* were calculated.

### Antibodies, Western Blotting

Rac activation assays were carried out as described previously [7]. Briefly, right lobes of the lungs from normoxia or hypoxia treated mice were isolated, washed 3 times with TBS, and homogenized with Mg^2+^ lysis/wash buffer (MLB, 25 mmol/L Tris, pH 7.5, 150 mmol/L NaCl, 1% Igepal CA-630, 10 mmol/L MgCl_2_, 1 mmol/L EDTA, 10% glycerol, 10 µg/ml leupeptin, 10 µg/ml aprotinin, 1 mmol/L sodium pervanadate, 1 mmol/L phenylmethylsulfonyl fluoride) on ice. These lysates were used for standard Western blotting and Rac activation assays. Lung lysates (in total, samples from 6 mice per genotype per condition) and PASMCs lysates in MLB (from triplicate wells and two independent experiments) were incubated with recombinant Pak1-GST PBD precoupled with glutathione-agarose beads at 4°C with rotation for 1 hour. Beads were washed 3 times with MLB and resuspended in SDS-sample buffer. Lysates to measure total Rac1 were collected before performing the Rac activation assay. Immunoblotting was performed with anti-Rac1 antibodies (1∶1000, Cytoskeleton). Anti p38 and p-p38 antibodies (1∶500) were from Cell Signaling. Abr antiserum (1∶200) has been previously described. [8] Bcr (N-20; 1∶500) antibodies were from Santa Cruz and GAPDH antibodies (1∶5000) from Millipore. Anti-phosphotyrosine antibodies were obtained from BD Biosciences. Western blot results were quantified using Un-Scan-It software (Silk Scientific, Orem, UT) and analyzed as the ratio of phosphorylated p38 MAPK to total p38 MAPK or of GTP-bound Rac1 to total Rac. Western blot analysis was done with Abr antiserum (1∶200) [8] and Bcr (N-20)(1∶500, Santa Cruz) or GAPDH antibodies (1∶5000, Millipore).

### ELISA

The IL-6 levels in the serum and lungs of mice and PASMCs supernatants were measured by Enzyme-Linked Immunosorbent Assay (ELISA) according to the manufacturer's instructions (eBioscience).

### Statistical Analysis

Statistical analysis was performed with SPSS 13.0 software (Chicago, USA). Data are expressed as mean ±SD. One way-ANOVA was used to compare the differences between groups, followed by the Student-Newman-Keuls post-hoc analysis. Statistical significance was considered when p<0.05.

## Supporting Information

Figure S1 **Emphysema and airway remodeling in normoxia- and hypoxia-exposed mice. A,** Representative H&E stained lung sections of the indicated genotypes showing alveoli and bronchiolar walls. Bars, 25 µm. **B-C,** Quantification of **B**, mean linear intercept (MLI), and **C,** alveolar area. n.s., not significant.
(JPG)

Figure S2 **Treatment of hypoxia- and normoxia-exposed mice with Z62954982. A,** Right ventricle hypertrophy assessed by ratio of (RV/LV+S) from the hearts of normoxic and hypoxic *wt* and *abr−/−* mice treated with Z62954982 at 10 mg/kg every other day or at 20 mg/kg daily for 3 wks. Control mice were administered i.p. with equal amount of vehicle. Bars, mean+SEM. n = 3–4 mice/group. *, p<0.05, data were analyzed by one way ANOVA. **B–C**, Western blot analysis of representative samples (**B**) and quantification (**C**) of activated Rac1 in mice treated with 10 mg/kg Z62954982. n = 3 samples/group. Lysates were generated 1 hr after injection of the drug.
(TIF)

Figure S3 **Lack of toxicity of Z62954982.** **A**, representative H&E-stained liver sections of the indicated mice treated with 10 mg/kg every second day. **B**, analysis of total bone marrow cellularity and myeloid cell percentages of mice exposed to hypoxia and treated every day with 20 mg/kg drug or with vehicle. *p<0.05, n = 3 mice/group.
(TIF)
