# Supplementary figures and images for: Lack of Bcr and Abr Promotes Hypoxia-Induced Pulmonary Hypertension in Mice
Source: PLoS One. 2012 Nov 12;7(11):e49756. doi: 10.1371/journal.pone.0049756 (PMC3495860; doi:10.1371/journal.pone.0049756)

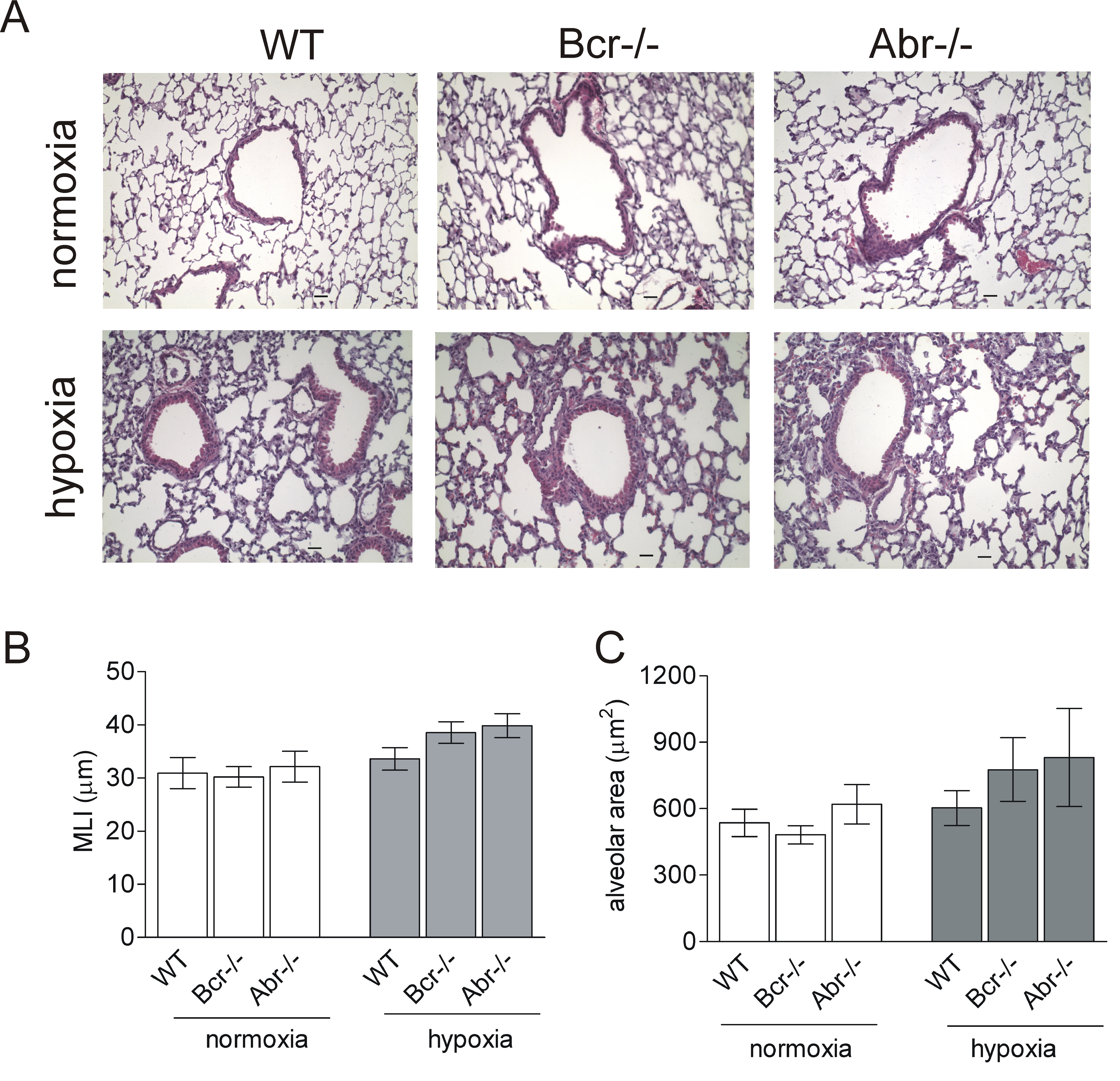

Supplement: Figure S1 — Emphysema and airway remodeling in normoxia- and hypoxia-exposed mice. A, Representative H&E stained lung sections of the indicated genotypes showing alveoli and bronchiolar walls. Bars, 25 µm. B-C, Quantification of B, mean linear intercept (MLI), and C, alveolar area. n.s., not significant. (JPG) [file pone.0049756.s001.jpg]

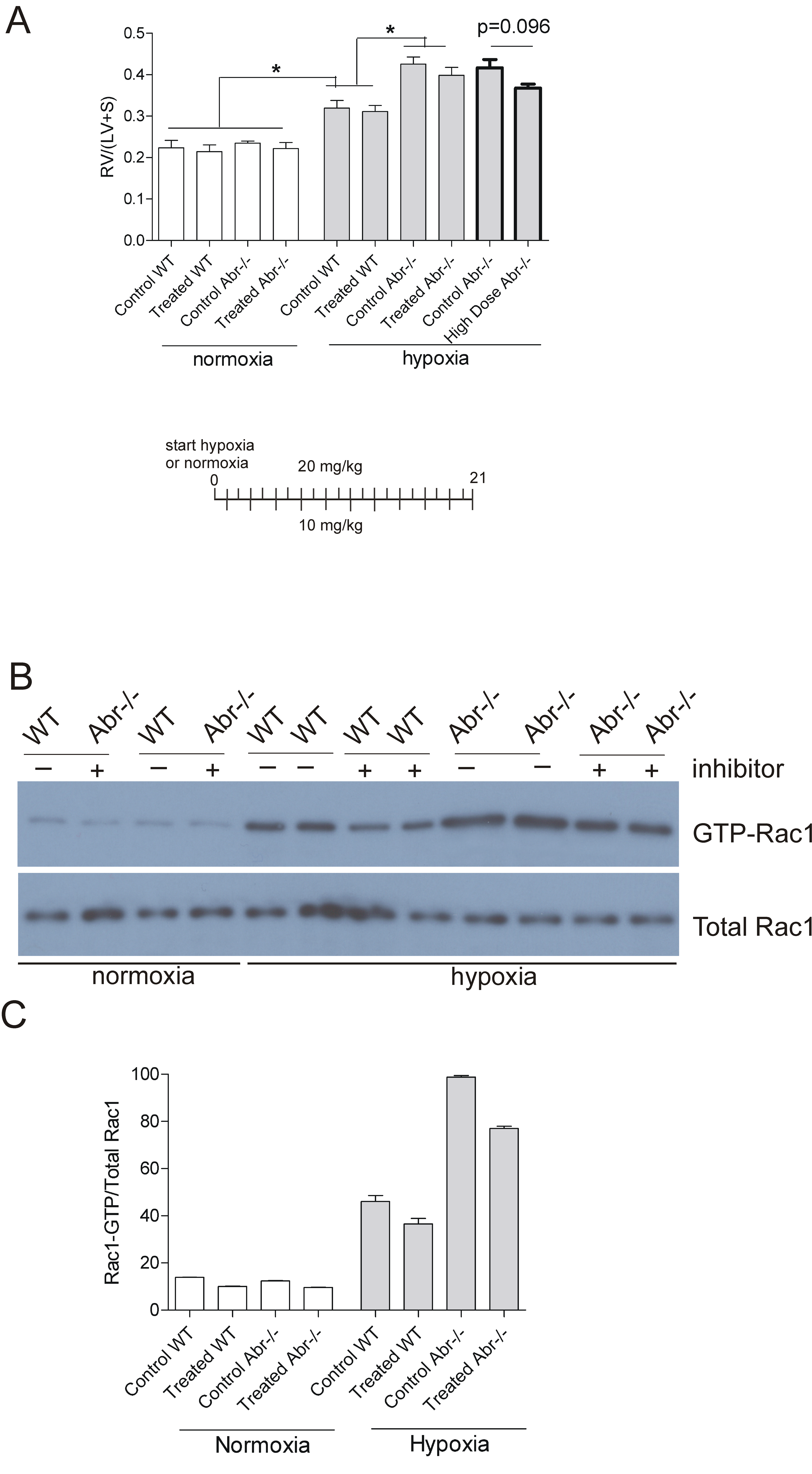

Supplement: Figure S2 — Treatment of hypoxia- and normoxia-exposed mice with Z62954982. A, Right ventricle hypertrophy assessed by ratio of (RV/LV+S) from the hearts of normoxic and hypoxic wt and abr−/− mice treated with Z62954982 at 10 mg/kg every other day or at 20 mg/kg daily for 3 wks. Control mice were administered i.p. with equal amount of vehicle. Bars, mean+SEM. n = 3–4 mice/group. *, p<0.05, data were analyzed by one way ANOVA. B–C, Western blot analysis of representative samples (B) and quantification (C) of activated Rac1 in mice treated with 10 mg/kg Z62954982. n = 3 samples/group. Lysates were generated 1 hr after injection of the drug. (TIF) [file pone.0049756.s002.tif]

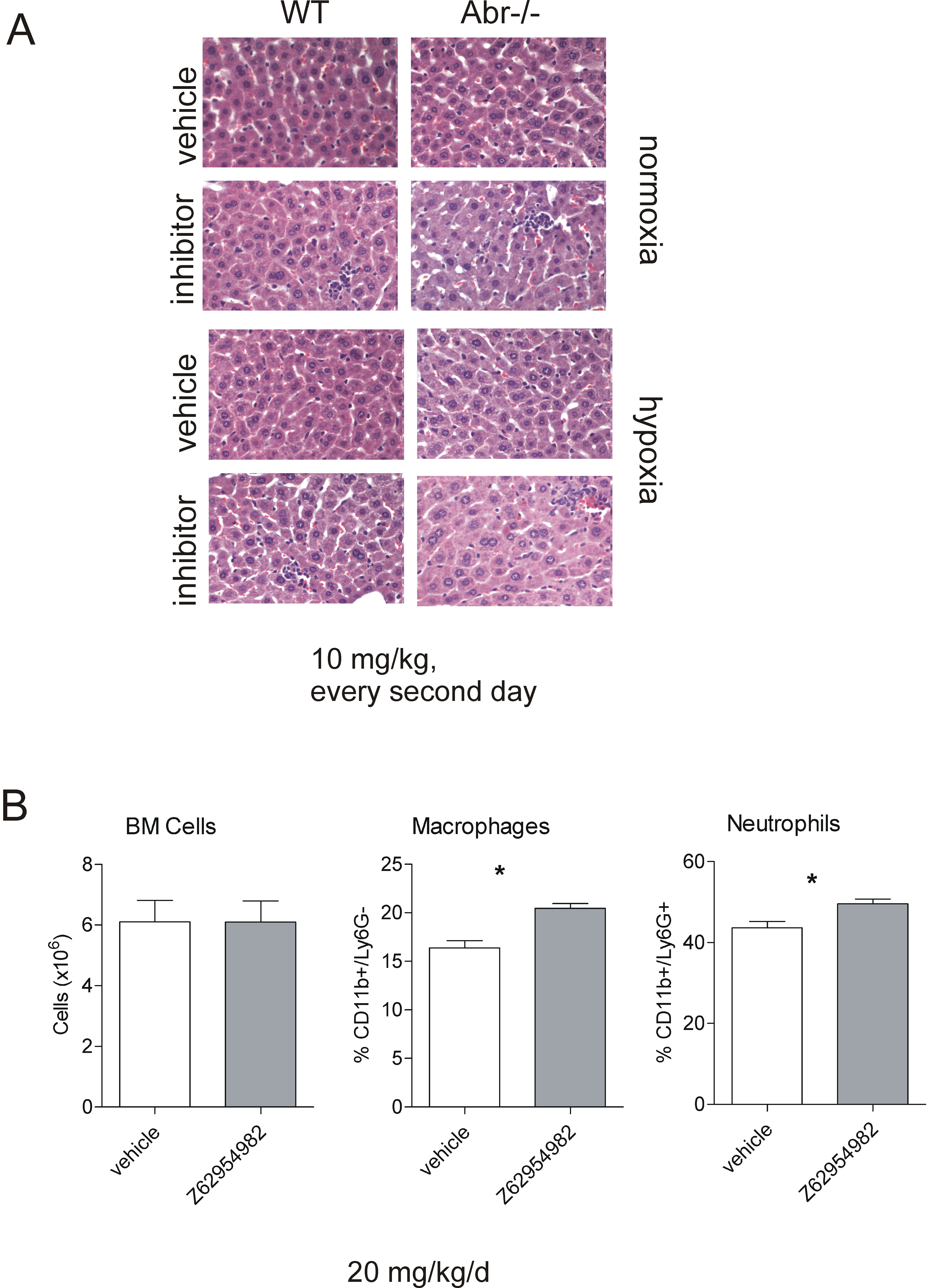

Supplement: Figure S3 — Lack of toxicity of Z62954982. A, representative H&E-stained liver sections of the indicated mice treated with 10 mg/kg every second day. B, analysis of total bone marrow cellularity and myeloid cell percentages of mice exposed to hypoxia and treated every day with 20 mg/kg drug or with vehicle. *p<0.05, n = 3 mice/group. (TIF) [file pone.0049756.s003.tif]
